# Harmonizing Outcomes for Genomic Medicine: Comparison of eMERGE Outcomes to ClinGen Outcome/Intervention Pairs

**DOI:** 10.3390/healthcare6030083

**Published:** 2018-07-13

**Authors:** Janet L. Williams, Wendy K. Chung, Alex Fedotov, Krzysztof Kiryluk, Chunhua Weng, John J. Connolly, Margaret Harr, Hakon Hakonarson, Kathleen A. Leppig, Eric B. Larson, Gail P. Jarvik, David L. Veenstra, Christin Hoell, Maureen E. Smith, Ingrid A. Holm, Josh F. Peterson, Marc S. Williams

**Affiliations:** 1 Genomic Medicine Institute, Geisinger, Danville, PA 17822, USA; Jlwilliams3@geisinger.edu; 2 Departments of Pediatrics and Medicine, Columbia University, New York, NY 10025, USA; wkc15@cumc.columbia.edu; 3 Irving Institute for Clinical and Translational Research, Columbia University, New York, NY 10025, USA; avf2117@cumc.columbia.edu; 4 Department of Medicine, Division of Nephrology, Columbia University, New York, NY 10025, USA; kk473@cumc.columbia.edu; 5 Department of Biomedical Informatics, Columbia University, New York, NY 10025, USA; chunhua@columbia.edu; 6 Children’s Hospital of Philadelphia, Philadelphia, PA 19104, USA; connollyj1@chop.edu (J.J.C.); harrm@email.chop.edu (M.H.); hakonarson@email.chop.edu (H.H.); 7 Perelman School of Medicine, University of Pennsylvania, Philadelphia, PA 19104, USA; 8 Genetic Services, Kaiser Permanente of Washington, Seattle, WA 98101, USA; leppig.k@ghc.org; 9 Kaiser Permanente Washington Health Research Institute, Seattle, WA 98101, USA; larson.e@ghc.org; 10 Departments of Medicine (Medical Genetics) and Genome Sciences, University of Washington, Seattle, WA 98195, USA; gjarvik@medicine.washington.edu; 11 Department Pharmacy, University of Washington, Seattle, WA 98195, USA; veenstra@uw.edu; 12 Center for Genetic Medicine, Northwestern University, Chicago, IL 60611, USA; christin.hoell@northwestern.edu (C.H.); m-smith6@northwestern.edu (M.E.S.); 13 Division of Genetics and Genomics, Boston Children’s Hospital, and Department of Pediatrics, Harvard Medical School, Boston, MA 02115, USA; Ingrid.Holm@childrens.harvard.edu; 14 Departments of Biomedical Informatics and Medicine, School of Medicine, Vanderbilt University, Nashville, TN 37232, USA; josh.peterson@Vanderbilt.Edu

**Keywords:** genomics, genomic medicine, health outcomes, evidence, standards, eMERGE, ClinGen, precision public health

## Abstract

Genomic medicine is moving from research to the clinic. There is a lack of evidence about the impact of genomic medicine interventions on health outcomes. This is due in part to a lack of standardized outcome measures that can be used across different programs to evaluate the impact of interventions targeted to specific genetic conditions. The eMERGE Outcomes working group (OWG) developed measures to collect information on outcomes following the return of genomic results to participants for several genetic disorders. These outcomes were compared to outcome intervention pairs for genetic disorders developed independently by the ClinGen Actionability working group (AWG). In general, there was concordance between the defined outcomes between the two groups. The ClinGen outcomes tended to be from a higher level and the AWG scored outcomes represented a subset of outcomes referenced in the accompanying AWG evidence review. eMERGE OWG outcomes were more detailed and discrete, facilitating a collection of relevant information from the health records. This paper demonstrates that common outcomes for genomic medicine interventions can be identified. Further work is needed to standardize outcomes across genomic medicine implementation projects and to make these publicly available to enhance dissemination and assist in making precision public health a reality.

## 1. Introduction

Genomic medicine is defined by the National Human Genome Research Institute (NHGRI) as, “an emerging medical discipline that involves using genomic information about an individual as part of their clinical care (e.g., for diagnostic or therapeutic decision-making) and the health outcomes and policy implications of that clinical use” [1]. Prior research has demonstrated that genomic medicine has promise for improving health outcomes. As a result, it is beginning to emerge into the clinical practice for selected indications including pharmacogenomics [2], precision oncology [3], and diagnosis of complex conditions suspected be genetic [4]. Large-scale research programs such as the All of Us program funded by the United States National Institutes of Health (NIH) [5] and smaller private clinical research programs [6,7] are beginning to explore the integration of genomic information with other health information to assess the impact on patient outcomes that, it is hoped, will ultimately result in more programs in precision public health.

Several barriers to the implementation of genomic medicine have been identified [8]. One of the most important of these is the lack of evidence of the clinical utility of the interventions. Stated another way, while there is strong evidence about the association of genomic variation with genetic disorders, there is, with few exceptions, inadequate information about the impact on outcomes (both positive and negative) of implementing genomic medicine into clinical care [9,10]. This lack of evidence results in a reluctance of healthcare systems to invest in and payers to reimburse for genomic medicine interventions. There is a general agreement that evidence of the impact of genomic medicine on health outcomes must be generated. There are many barriers to the generation of evidence [9,10], one of which is the lack of agreed-upon outcomes to measure the impact of conditions of interest.

The NHGRI has funded several large collaborations to study genomic medicine in clinical care. These include, but are not limited to, the Implementing Genomics in Practice (IGNITE) network [11], the Clinical Sequencing Evidence-Generating Research (CSER) consortium [12], and the Electronic Medical Records and Genomics (eMERGE) network [13]. All three of these groups have a workgroup tasked to develop outcomes for site-specific and network projects. While these groups have worked to harmonize outcomes within each project, it was not until 2017 that an effort started to try to harmonize outcomes across these and potentially other NHGRI-funded projects. This was initially accomplished by creating formal liaisons between each of the respective outcomes groups, and by holding joint meetings between the networks/consortium [14]. While this has resulted in some convergence, the differences between the projects and the lack of alignment of the project timelines have hindered the agreement on a standard set of outcomes across the three networks.

eMERGE is in its third phase of funding. The focus of this phase is the return of genomic results to participants [15]. A total of just over 25,000 participants will be sequenced on a next-generation sequencing platform, eMERGEseq, that contains 109 genes and a number of single nucleotide variants, including pharmacogenomic variants that may also be returned to participants [16]. The eMERGE Outcomes Working Group (OWG) was tasked to develop outcome measures for a set of genetic disorders for which the associated genes would be interrogated by sequencing. The OWG identified another NHGRI-funded project, the Clinical Genome Resource (ClinGen) [17] that had a relevant activity that could be used to move outcomes harmonization forward. Herein we report the results of a comparison between the eMERGE-defined outcomes and the ClinGen outcome intervention pairs.

## 2. Materials and Methods

eMERGE network sites represented on the OWG selected a disorder(s) for which their site developed clinical outcome measures. The outcomes were organized into three categories, process outcomes, intermediate outcomes, and health outcomes (Table 1). While health outcomes are of the greatest interest, the relatively short project timeline necessitated reliance on the process and intermediate outcomes for which a chain of evidence exists relating them to health outcomes of interest. Sites developed outcomes using their own approach, with the expectation that any proposed outcomes would have evidence of its relevance to clinical care. Emphasis was given to outcomes that were related to published clinical and practice guidelines where available. Once the draft outcomes were developed, they were presented to the OWG for discussion and revisions. The penultimate draft was submitted to the eMERGE coordinating center that, under the direction of one of the OWG co-chairs (JP), was tasked to develop the outcomes into a collection tool that could be created in REDCap [18] using a standard format. The coordinating center worked with the individual sites to create the final version of the outcomes.

The ClinGen Actionability Working Group (AWG) was tasked to assess the relative actionability of returning a genomic variant identified in an asymptomatic patient undergoing next-generation sequencing [19]. This was to be accomplished through four activities:

1. Develop rigorous and standardized procedures for categorically defining “clinical actionability”; a concept that includes a known ability to intervene and thereby avert a poor outcome due to a previously unsuspected high risk of disease
2. Nominate genes and diseases to score for “clinical actionability”
3. Produce evidence-based reports and semi-quantitative metric scores using a standardized method for nominated gene-disease pairs
4. Make these reports and actionability scores publicly available to aid broad efforts for prioritizing those human genes with the greatest relevance for clinical intervention.

The AWG has developed a set of outcome intervention pairs [20] that have been scored using a standardized approach informed by evidence-based summaries as described in a methods paper from 2016 [21]. The published outcome intervention pairs table represents those that have been scored by the AWG. The evidence summary also contains interventions and outcomes that were not formally scored. Both the table and the associated evidence summary were reviewed to completely ascertain the interventions and outcomes that had been reviewed by the AWG.

For the comparison, each site participating in the exercise compared the set of outcomes developed for the disorder in eMERGE to the corresponding outcome intervention pair published on the AWG website. If the eMERGE outcome was represented in the scored AWG outcome intervention pair, it was categorized as concordant. If it was not represented in the scored AWG outcome intervention pair, but was noted in the evidence summary, it was also categorized as concordant with the annotation that it did not cross the threshold for scoring by the AWG. If the outcome was not present in either the scored list or evidence summary, it was categorized as discordant. Conversely, if an outcome intervention was present on the AWG scored list, but not represented as an eMERGE outcome, it was also categorized as discordant. The evidence summaries were not comprehensively reviewed for outcomes to compare to eMERGE outcomes.

The sites’ comparisons were compiled and reviewed by one of the authors (MSW) who also independently compared the eMERGE outcomes to the AWG outcome intervention pairs. No differences were noted between the sites’ scores and the second review for the AWG outcome intervention pairs. A few outcomes were identified in the evidence summaries that had not been scored by the sites, and these were added to the comparison table. The final comparison table was reviewed and approved by all the authors.

## 3. Results

A total of 12 disorders were scored (Table 2 and Table 3). The full comparison table with all defined eMERGE outcomes for each disorder is provided in the supplemental materials. Three gene/variant disorder pairs with outcomes defined by eMERGE do not have an AWG actionability score or evidence summary. *CFTR*/Cystic Fibrosis is being returned by eMERGE but has not yet been evaluated by the ClinGen AWG. While adult familial hypercholesterolemia (FH associated with the genes *LDLR*, *APOB*, and *PCSK9*) has been evaluated by both the OWG and AWG, FH in the pediatric population has only been evaluated by the OWG. This is because ClinGen initially focused on conditions in the adult population. However, this year, a pediatric AWG is being convened by ClinGen and one of their first conditions to evaluate will be pediatric FH. Finally, eMERGE is studying a large, well-characterized copy number variant (CNV) at chromosome 22q11.2 that encompasses many genes. The AWG is only looking at single gene-disorder associations at present.

Of the remaining nine gene(s)-disorder pairs defined by eMERGE, five had equivalent definitions from the AWG, while four had some differences which raised interesting issues that impacted the comparison. These two groups will be discussed separately.

The five disorders with equivalent definitions from both groups and the associated genes are presented in Table 2. It should be noted that the eMERGE project is only returning results from two genes that are associated with breast and/or ovarian cancer risk (*BRCA1* and *BRCA2*). Three genes with evidence for association with breast cancer are on the eMERGEseq platform (*ATM*, *CHEK2*, *PALB2*), but were not used to develop outcomes. These have been scored by the AWG but had much lower actionability scores than *BRCA1* and *BRCA2*; therefore, they were excluded from the comparison for the purposes of this study.

Comparing AWG scoring to the eMERGE outcomes list demonstrates significant concordance. Only two of the outcome intervention pairs scored by AWG was not present in the eMERGE outcomes. Both of these represented health outcomes (diagnosis of tumors and/or lymphangioleiomyomatosis (LAM) in the tuberous sclerosis complex (TSC) and high cholesterol in adult FH. For the latter, lipid values will be obtained from EHR review so a determination can be made as to whether a participant who has been tested is at a goal. Thus, while this is not explicitly represented in the eMERGE outcomes, it should be added given the robust association between low-density lipoprotein cholesterol (LDLC) and cardiovascular events [22,23,24]. For the TSC health outcomes, eMERGE will be capturing information about the prior diagnosis of sub-ependymal giant astrocytoma (SEGA), other TSC-associated non-SEGA tumors, and LAM. It is also possible that the diagnostic evaluation prompted by the genomic result could lead to a diagnosis of one of the conditions. However, given the short time period of the eMERGE project, a long-term longitudinal follow-up is not feasible, in contrast to the AWG score, which is meant to inform interventions over a patient’s lifetime.

While most of the eMERGE outcomes are not represented in the AWG scored outcome intervention pairs, most are discussed in the evidence review that accompanies the scored pairs. The AWG methodology does not score all possible outcome intervention pairs, rather it focuses on those interventions that have the strongest impact on the most important health outcomes of interest.

Hereditary breast and ovarian cancer syndrome (HBOC), associated with *BRCA1/2*, illustrates an interesting difference in the OWG and AWG approaches. The eMERGE OWG developed outcomes for HBOC as a whole, while the AWG has organized this around the two primary cancer types, breast, and ovarian and associated gynecologic cancers. This is logical as the outcome intervention pairs for the two types of cancers are quite different. This is not incompatible with the eMERGE outcomes, and Table 2 reflects how the outcomes can be separated to allow comparison.

A more important difference in the approach between the two groups is illustrated in Table 3. The four disorders represented, cardiomyopathy, inherited arrhythmogenic disorders, aortopathies, and colorectal cancer (CRC) predisposition illustrate the tension between pragmatic decisions to reduce the burden to collect outcomes of interest at the expense of capturing outcomes that are specific to individual disorders lumped within the overarching category of disorders. Some of these differences are clinically significant as discussed below.

### 3.1. Colorectal Cancer Predisposition

The eMERGE outcomes combine two disorders, Lynch syndrome (LS) and the rarer familial adenomatous polyposis (FAP), while these are scored separately by the ClinGen AWG. There is good concordance between eMERGE and the AWG scored intervention outcome pairs. One significant difference is in FAP, for which the AWG does not score CRC surveillance. Review of the evidence summary presents the rationale that the polyp burden reduces the effectiveness of surveillance. The outcome intervention pair scored by the AWG for FAP is colectomy to prevent CRC. This is consistent with the clinical guidelines for FAP [25], although this recommendation may not be as relevant for patients with attenuated FAP, as they have fewer polyps than FAP (hundreds vs. thousands). Colectomy is listed as an option for reducing the risk of CRC in patients with LS, but is generally not indicated due to the effectiveness of routine colonoscopy in prevention. Another difference between FAP and LS is that the non-CRC tumors differ and occur at a higher frequency in LS. This necessitates different screening approaches which are detailed in the AWG evidence reports. Finally, the AWG evidence reports also discuss the use of aspirin (LS) and non-steroidal anti-inflammatory drugs other than aspirin (FAP) to reduce the CRC risk. These should be considered for inclusion in the eMERGE outcomes.

### 3.2. Aortopathies

The OWG developed outcomes to accommodate all disorders that could result in aortic root dilation and other arteriopathies. The AWG divided these into arterial tortuosity syndrome (associated with variants in *SLC2A10*), and Familial Thoracic Aortic Aneurysms and Dissections (FTAAD associated with seven genes-Table 3). The AWG scored each of these FTAAD genes separately, although the evidence summary was the same for all seven genes. The actionability scores for the seven gene-disorder pairs were identical. As with CRC, there was very good concordance between the eMERGE outcomes and the AWG scored outcome intervention pairs. Indeed, the only discrepancies were recommendations for avoidance of contact sports and evaluation by an ophthalmologist, both present as a scored recommendation for arterial tortuosity syndrome, present in the evidence summary for FTAAD but not scored, and absent from eMERGE. Given that many of these disorders have associated ophthalmologic findings, this should be considered as an outcome by the eMERGE OWG. Recommendations to avoid activities such as contact sports are difficult to extract from medical records, so they were not considered for practical considerations.

There is one other issue with the aortopathies that complicates outcome development. There are two multiple malformation syndromes that can be seen in patients with variants in some of these genes, the Marfan and Loeys-Dietz syndromes. This complexity was acknowledged by the ClinGen AWG, as both disorders have been scored as separate entities. These syndromes are associated with many other medical issues; however, the scored outcome intervention pairs are concordant with the recommendations for aortic root dilation represented in arterial tortuosity syndrome and FTAAD. However, the evidence summary goes into much more detail about the other medical issues associated with these syndromes. The eMERGE OWG recognizes this issue and it is anticipated that a targeted clinical evaluation will occur in conjunction with the return of results.

### 3.3. Cardiomyopathies

The eMERGEseq platform has 14 genes associated with three forms of cardiomyopathy: dilated, hypertrophic, and arrhythmogenic right ventricular (ARVC). One form was developed to capture outcomes for all three disorders. The ClinGen AWG scored each of the three disorders separately, and further scored each of the five ARVC genes separately, although as with FTAAD, the scores were identical for each of the five genes. The major risk for all three of these disorders is sudden death, and this health outcome is common across all the conditions. Related to this, an implantable cardiac defibrillator (ICD) is also present across all conditions. Not surprisingly, given the differences in the clinical course of these three conditions, beyond sudden cardiac death and ICD, there is a considerably more difference in the other outcomes. Most of these differences appropriately reflect the clinical differences between the conditions. There is only one AWG recommendation that is not reflected in the OWG outcomes. A creatine kinase determination is recommended for dilated cardiomyopathy associated with variants in *DMD*. However, *DMD* is not included on the eMERGEseq platform, explaining this difference. One gene associated with dilated cardiomyopathy, *LMNA*, is associated with several other disorders. One of them is Emery-Dreifuss Muscular Dystrophy (EDMD), which was scored separately by the AWG. There were other outcome intervention pairs scored for EDMD in addition to those related to cardiomyopathy. The eMERGE network decided that it would only return variants in *LMNA* associated with dilated cardiomyopathy, so outcomes for the other disorders were not considered. One other issue with the cardiomyopathies reviewed by the AWG is that variants in *TNNT2* can cause either dilated or hypertrophic cardiomyopathy. This pleiotropy will be more of an issue in the next group of disorders.

### 3.4. Inherited Arrhythmias

The eMERGEseq platform has four genes associated with three inherited arrhythmogenic disorders: Brugada syndrome, catecholaminergic polymorphic ventricular tachycardia (CPVT), and Romano-Ward Long QT syndromes (LQT). As with the cardiomyopathies, the major risk is for sudden death. This health outcome is represented across all conditions. ICD is an AWG recommendation for two of the three conditions. CPVT is the exception given the effectiveness of the beta-blockade to prevent sudden cardiac death in this disorder. There are numerous differences between the OWG outcomes and the AWG that reflect the differences in the conditions. The most notable absence from the eMERGE outcomes were medications to avoid in each condition. The AWG evidence reports provide detailed lists of medications and other substances to avoid as they can provoke abnormal cardiac rhythms. These are important to document and should be considered in addition to the eMERGE outcomes, as the documentation of medications associated with adverse events are relatively easy to find on the chart review.

As noted with *TNNT2* previously, one gene (*SCN5A*) is associated with two different arrhythmogenic disorders: Brugada syndrome and LQT3. There are several unique aspects to disorders associated with variants in *SCN5A.* For patients with Brugada syndrome, a trial of therapy with sodium channel blockers is indicated. The recommended anti-arrhythmic drug is quinidine. Both recommendations are specific only for the arrhythmogenic disorders associated with variants in *SCN5A*. For LQT3, the treatment with beta-blockers is not indicated as these have been shown to be ineffective in this condition. These findings argue persuasively for outcomes that are not only condition specific but gene and potentially even variant specific when appropriate.

## 4. Discussion

The results of this study show that it is possible to compare outcomes from two projects despite differences in the project objectives and methods. The important finding is that outcomes that are represented across multiple projects can be prioritized to harmonize the outcome definitions and develop guidance for their collection. This will facilitate the collection of prioritized outcomes from a wider set of research projects and clinical implementations, allowing evidence to accumulate at a faster rate to support clinical use. An example of the power of this type of approach for a genetic condition is cystic fibrosis (CF). Certified CF centers who receive funding from the CF Foundation are required to collect and submit many standard outcome measures. The outcomes are compared across sites and opportunities to improve care are identified, followed by implementation at the centers. This approach, which is also being used in other settings, has resulted in a dramatic improvement in multiple outcomes of interest for patients with CF [26]. The hope is that similar improvements in care could be realized across the many conditions for which genomic information can be used to inform care.

While there was generally good agreement for the high-level outcomes across the various conditions, there are some significant differences—the highlighting of which could inform further efforts to harmonize outcomes. eMERGE and ClinGen have very different objectives. The eMERGE network is studying the impact of implementation of genomic information into clinical care. To fully understand this impact, the outcomes are much more granular and detailed to allow chart abstractors to identify relevant information from the EHR. For example, in the cardiomyopathies (Table 3), process outcomes include five different interventions that assess the cardiac conduction system and two imaging modalities. The ClinGen scored outcome/intervention pairs only list one assessment of the cardiac conduction system and one imaging modality, and that was only for dilated cardiomyopathy. This is understandable as the scored pairs represent the results of the evidence synthesis that identifies the interventions and outcomes that drive clinical actionability, the key objective for ClinGen—a much different objective compared to eMERGE. Nonetheless, most of the eMERGE outcomes were identified in the ClinGen evidence reviews, although the reviews identified a few outcomes not included in the eMERGE OWG outcomes that are worthy of consideration for inclusion. Additionally, the AWG scored some gene-disorder pairs that, while on the eMERGEseq platform, are not being routinely returned. If the OWG proceeds with outcomes development for these genes, the AWG outcome intervention pairs and evidence summary will be used to inform the process.

A more complex issue is illustrated by the conditions in Table 2 and Table 3, that is, how best to map outcomes for separate but related disorders. While it may be desirable to create outcomes specific for each disorder within a category, the time and effort required to do this are significant. Therefore, the eMERGE OWG opted to develop one outcome form for an overarching disorder category that encompasses multiple conditions. While this reduces the resources needed to create the outcome forms and simplifies the work for the chart abstractor, it will require more effort by the OWG after the abstraction to map the outcomes that are specific to the relevant disorder in order to determine whether appropriate condition-specific management goals were achieved. Challenges with this issue are also evident in the ClinGen AWG scoring as some conditions lump all genes under one disorder (e.g., familial hypertrophic cardiomyopathy), while others have a separate score for each gene (e.g., FTAAD, ARVC). In these examples the scored outcome intervention pairs are identical across the different genes, raising the question as to the value added from this approach. In contrast, the three LQT disorders have different interventions based on the causal gene, supporting separate scoring of the outcome intervention pair. A further complication involves a pleiotropy of disorders associated with variants in the same gene. The issues with *SCN5A* and *LMNA* described previously illustrate the challenges of developing outcomes for disorders associated with variants in these genes. The most precise solution would be to develop outcomes based on the established genotype-phenotype correlations, but this further increases the complexity. This issue has led to the creation within ClinGen of the Lumping and Splitting Working Group (LSWG) [27]. The goal of the LSWG is to engage with a broad range of stakeholders to gather input “… to coordinate disease classification and categorization in order to harmonize disease categorization and classification for the greater community”. The work product from this group will be incorporated into the ongoing efforts for outcomes harmonization.

**Figure 1:**
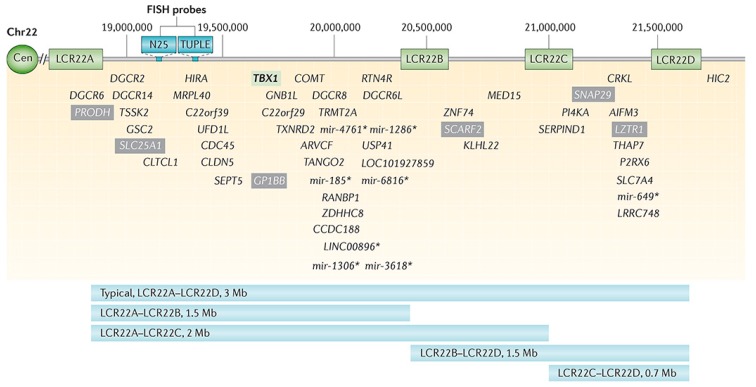
The depiction of the chromosome 22q11.2 deletion including the deleted genes and variations of the common deletions reported.

Chromosome 22q11.2 deletion syndrome (22q11.2DS) is the most common chromosomal microdeletion disorder with approximately 3.0 million base pairs deleted (ranging from 0.7–3.0 Mb) resulting in a loss of ~90 known or predicted genes, including 46 protein-coding genes and 7 microRNAs, 10 non-coding RNAs, and 27 pseudogenes (Figure 1) [28]. The 22q11.2DS results most commonly from de novo non-homologous meiotic recombination events occurring in approximately 1 in every 1000 fetuses and 1 in 2000 live births. About 4% of infants with 22q11.2DS succumb to it, while cardiac defects, hypocalcemia, and airways disease are risk factors for early death, with the median age of death at 3–4 months. However, most individuals with 22q11.2DS survive well into adulthood, at which time approximately 50% of them develop schizophrenia.

While ClinGen (currently) makes no recommendations with respect to 22q11.2DS we note the syndrome has become a model for understanding rare and frequent congenital anomalies such as heart defects, medical conditions including immunodeficiency, allergies, asthma, and psychiatric and developmental differences, which may provide a platform into better understanding these phenotypes, while affording opportunities for translational strategies across the lifespan for both patients with 22q11.2DS and for those with these associated features in the general population. The diverse phenotype and outcomes of nearly every organ system make this population valuable for understanding the variables that impact on the manifestations of the deletion, which is relatively consistent from person to person.

The eMERGESeq panel captures six SNPs (five in the *COMT* gene and one flanking the region), which can be used to capture 22q11.2DS, while existing genotype data can be readily used to detect the syndrome. Current efforts aim at assessing the prevalence of 22q11.2DS in respective eMERGE cohorts, and to determine a health outcome across multiple organ systems and outcome measures as available.

We are using PennCNV and XHMM to derive CNVs from eMERGESeq data, as well as existing array data. Data will be returned to participating sites for outcome evaluation of relevant phenotypes (e.g., heart defects, immunodeficiency, allergy, asthma, psychiatric, and developmental differences) and for additional validation, if required.

This study represents a pilot to assess the feasibility of harmonizing outcomes across two notable research projects. As such the results are descriptive and limited to the two projects assessed. The study did not include the evaluation of outcomes for any clinical genomic medicine implementation projects. However, one eMERGE site reports the genomic results on a large scale in a clinical research setting [7]. Institutional authors (MSW, JLW), in conjunction with the Genetic Screening and Counseling Program at the institution, have aligned the eMERGE and institutional outcomes for the disorders shared in common between the two efforts (data not shown). The availability of the outcomes from eMERGE aided in the prioritization of the institutional outcomes, while input from the authors, both of whom are members of the eMERGE OWG, influenced the outcome definitions for the OWG. This illustrates that the harmonization of outcomes is not only feasible but may represent a generalizable approach. Mapping outcomes to standardized, structured terminologies such as the International Classifications of Disease (ICD) or the Systematized Nomenclature of Medicine-Clinical Terms (SNOMED-CT) would facilitate generalizability and reduce the reliance on manual collection, although it is important to note that many critical outcomes are not currently represented as structured data so some manual review will be required. It is possible that outcome “algorithms” could be developed. These would be similar to phenotyping algorithms that eMERGE has developed, disseminated across multiple healthcare and electronic health record systems and made publicly available through the Phenotype Knowledgebase-PheKB. [29] This could further reduce, although not eliminate, the burden of manual review.

Another limitation of this study was the outcomes and process measures such as cost, reimbursement, institutional visibility, access, etc., which also play a role in decisions about implementation were not assessed. We also did not focus on patient-centered outcomes, which are not always aligned with health or other outcomes. Measuring outcomes from the perspective of the patient has been identified as a deficiency in much medical research as evidenced by the creation of the Patient-centered Outcomes Research Institute (PCORI) in 2010 [30]. The PCORI vision statement (“patients and the public have the information they can use to make decisions that reflect their desired health outcomes”) emphasizes that part of precision medicine is understanding what outcomes the patient desires, which will vary from patient to patient. Patient engagement is a key part of the All of Us project [5], therefore, developing and harmonizing patient-centered outcomes for genomic medicine is important. Of interest, the NIH funded the development and harmonization of a large set of patient-centered outcome measures now included in the Patient-Reported Outcomes Measurement Information System (PROMIS^®^) [31] made available through the Department of Health and Human Services. These measures can be reviewed and revised as necessary to develop patient-reported outcomes for genomic medicine. This also illustrates that a process led by the NIH to collect and harmonize outcome measures across its portfolio of projects is a successful approach and can promote the use of standardized measures going forward.

## 5. Conclusions

The definition and harmonization of common outcomes to develop evidence and assess the value of genomic medicine implementation are needed to further the goals embodied in precision public health. The approach proposed in this study will be applied to other NHGRI-funded genomic implementation projects. The resulting outcomes will be made publicly available and their use will be encouraged for outcome measurement, collection, and research to accelerate the implementation of those interventions that demonstrate improved value.

## Figures and Tables

**Table 1:** The framework of outcomes for clinical implementation.

Outcome Type	Description	Examples
Process	The specific steps in a process that lead—either positively or negatively—to a particular health outcome	Lipid profile performed after the return of a pathogenic variant in *LDLR*, a gene associated with familial hypercholesterolemia
Intermediate	A biomarker associated—either positively or negatively—to a particular health outcome	An LDL cholesterol level at or below the target level of 100 mg/dL in response to interventions recommended based on presences of a pathogenic variant in *LDLR*
Health	Change in the health of an individual, group of people or population which is attributable to an intervention or series of interventions	Decrease in myocardial infarction, or cardiac revascularization procedures in response to interventions recommended based on presences of a pathogenic variant in *LDLR*

**Table 2:** Disorders with equivalent definitions from eMERGE and ClinGen.

Disorder	Genes	eMERGE Outcomes	AWG Scored O/I Pair	AWG Evidence Review
OTC Deficiency	*OTC*	**Process**		
		Metabolic Testing	No	Yes
		Metabolic Crisis Plan in EHR	No	No
		**Intermediate**		
		Low Protein Diet	Yes	
		Prescription for Nitrogen Scavenger	Yes	
		**Health**		
		Metabolic protocol applied during illness	Yes (Hyperammonemic encephalopathy)	
Tuberous Sclerosis	*TSC1*, *TSC2*	**Process**		
		Imaging studies	Yes	
		Assessment for LAM	Yes	
		**Intermediate**		
		Discontinuation of estrogen containing medications (F)	No	Yes
		Use of inhibitor of renin-aldosterone-angiotensin system as first line therapy for hypertension	No	No
		Avoid ACE inhibitor	No	No
		No	Use of mTOR inhibitor	
		**Health**		
		No	Development of SEGA, non-SEGA tumors, LAM	
HBOC (Breast)	*BRCA1*, *BRCA2*	**Process**		
		Breast Self-exam	Yes	
		Breast Imaging	Yes	
		Specialty Referral	No	Yes
		**Intermediate**		
		Risk reducing mastectomy	Yes	
		Selective estrogen receptor modulator	No	Yes
		Aromatase Inhibitor	No	No
		Discontinuation HRT	No	No
		**Health**		
		Breast Cancer	Yes	
		Vital Status	No	Yes
HBOC (Ovarian)	*BRCA1*, *BRCA2*	**Process**		
		Pelvic US	No	Yes
		CA 125	No	No
		Specialty Referral	No	Yes
		**Intermediate**		
		Prophylactic BSO or TAH/BSO	Yes	
		Oral Contraceptives	No	No
		**Health**		
		Ovarian, Fallopian, Peritoneal or Endometrial Cancer	Yes	
		Vital Status	No	Yes
Adult FH	*LDLR*, *APOB*, *PCSK9*	**Process**		
		Laboratory testing (lipid, CRP)	No	Yes
		Coronary CT angiogram	No	Yes
		Echocardiogram	No	Yes
		ECG	No	No
		Stress test	No	No
		Specialty Referral	No	No
		No		Cardiac Catheterization
		**Intermediate**		
		Lipid Lowering Therapy	Yes (statins)	High-intensity statins
		Aspirin	No	Yes
		Coronary revascularization	No	No
		No	High Cholesterol	
		**Health**		

**Table 3:** Disorders with differing definitions between eMERGE and ClinGen.

Disorder	Genes	eMERGE Outcomes	ClinGen Actionability Working Group					
**Colorectal Cancer**	*MLH1*, *MSH2*, *MSH6*, *PMS2*, *FAP*		**Lynch syndrome** (*MLH1*, *MSH2*, *MSH6*, *PMS2)*				**Familial Adenomatous Polyposis** (*FAP*)	
		**Process**	**Scored O/I Pair**		**Evidence Review**		**Scored O/I Pair**	**Evidence Review**
		Specialist Referral	No		No		No	Yes (Gastroenterology)
		**Intermediate**						
		CRC Screening	Yes				No	No
		Other cancer screening	Yes				No	Yes
		Familial Cascade Testing	No		Yes		No	Yes
		No					Colectomy	
		**Health**						
		CRC (Polyps, Hospitalization, Death)	Yes				Yes	
		Gynecologic cancer (endometrial, ovarian)	Yes				N/A	N/A
**Aortopathies**	*FBN1*, *TGFBR1/2*, *SMAD3*, *ACTA2*, *MYLK*, *MYH11*		**Arterial Tortuosity Syndrome** (*SLC2A10*)				**FTAAD** (*FBN1*, *TGFBR1/2*, *SMAD3*, *ACTA2*, *MYLK*, *MYH11)*	
		**Process**						
		Aortic Imaging	Yes				Yes	
		Magnetic Resonance Angiography	Yes				Yes	
		High risk pregnancy management	Yes				Yes	
		No	Recommendation to avoid contact sports				No	Yes
		No	Ophthalmologic eval				No	Yes
		**Intermediate**						
		Medication (beta-blocker, ARB)	Yes (both)				Yes (beta-blocker)	
		Prophylactic surgical intervention	No		Yes		No	Yes
**Cardiomyopathies**	*ACTC1*, *DSC2*, *DSG2*, *DSP*, *LMNA*, *MYBPC3*, *MYCH7*, *MYL2*, *MYL3*, *PKP2*, *TMEM43*, *TNNI3*, *TNNT2*, *TPM1*		**Dilated Cardiomyopathy** (*TNNT2*, *LMNA*, *DMD)*		**Hypertrophic Cardiomyopathy** (*ACTC1*, *CSRP3*, *MYBPC3*, *MYH7*, *MYL2*, *MYL3*, *PRKAG2*, *TNNI3*, *TNNT2*, *TPM1*)		**Arrhythmogenic Right Ventricular Cardiomyopathy** (*DSC2*, *DSG2*, *DSP*, *PKP2*, *TMEM43*)	
		**Process**	**Scored O/I Pair**	**Evidence Review**	**Scored O/I Pair**	**Evidence Review**	**Scored O/I Pair**	**Evidence Review**
		EKG	Yes		No	Yes	No	Yes
		Echocardiogram	Yes		No	Yes	No	Yes
		Holter Monitor	No	No	No	Yes	No	Yes
		Loop recorder	No	No	No	Yes	No	No
		Stress Test	No	No	No	Yes	No	No
		Electrophysiology Study	No	No	No	No	No	Yes
		Cardiac MRI	No	No	No	No	No	Yes
		**Intermediate**						
		Specialty Referral	Yes		No	Yes	No	No
		Medications	Yes		No	Yes	Yes	
		Implantable Defibrillator	Yes		Yes		Yes	
		Documentation of Activity Restriction	No	No	No	Yes	No	Yes
		**Health**						
		Sudden Cardiac Death	Yes		Yes		Yes	
		Reduce Heart Failure	Yes		No	No	No	No
**Inherited arrhythmias**	*KCNH2*, *KCNQ1*, *RYR2*, *SCN5A*		**Brugada syndrome** (*SCN5A*)		**Catecholaminergic polymorphic ventricular tachycardia** (*RYR2*)		**Romano-Ward Long QT syndromes** (*KCNH2*, *KCNQ1*, *SCN5A*)	
		**Process**						
		EKG	No	Yes	No	Yes	No	Yes
		Echocardiogram	No	No	No	No	No	No
		Holter Monitor	No	No	No	Yes	No	No
		Loop recorder	No	Yes	No	No	No	No
		Stress Test	No	No	No	Yes	No	No
		Electrophysiology Study	No	No	No	No	No	No
		Cardiac MRI	No	No	No	No	No	No
		Trial Sodium Channel Blocker	No	Yes	No	No	No	No
		Personal history of arrhythmias	No	Yes	No	Yes	No	Yes
		Specialty referral	No	Yes	No	No	No	No
		**Intermediate**						
		Symptoms suggestive of arrhythmia	No	Yes	No	Yes	No	Yes
		Medications	No	Yes (quinidine)	Yes		Yes (beta-blockers are ineffective for LQT3	
		Activity restriction	Yes		No	Yes	No	Yes
		ICD	Yes		No	No	Yes	
		**Health**						
		Sudden Cardiac Death	Yes		Yes		Yes	
